# Relationships Between Accuracy in Predicting Direction of Gravitational Vertical and Academic Performance and Physical Fitness in Schoolchildren

**DOI:** 10.3389/fpsyg.2018.01528

**Published:** 2018-08-23

**Authors:** Wayne Haynes, Gordon Waddington, Roger Adams, Brice Isableu

**Affiliations:** ^1^Research Institute for Sport and Exercise, Faculty of Health, University of Canberra, Canberra, ACT, Australia; ^2^Aix-Marseille Univ., PSYCLE, Aix-en-Provence, France

**Keywords:** perception of vertical, spatial processing ability, numerical cognition, STEM, academic performance, physical activity, cardio-respiratory fitness, pre-pubescent children

## Abstract

Enhanced levels of cardio-respiratory fitness (CRF) and physical activity (PA) are both positively associated with health and academic outcomes, but less is known about the spatial processing and perceptual components of PA. Perception of vertical (PV) is a spatial orientation ability that is important for PA, and is usually measured as relative accuracy in aligning an object to gravitational vertical against a tilted background. However, evidence is inconclusive regarding the relationship of PV to educational outcomes – most importantly, numeracy. Students were recruited from primary schools in the Australian Capital Territory. A group of 341 (females *n* = 162, mean age 11.3 years) children performed all the tests required for this study. A computerised rod and frame test of PV employing a small (20°) visual angle was administered, and socio-economic status (SES), national education test results (NAPLAN, 2010), and CRF and PA data were collected. Correlation and hierarchical regression analysis were used to examine the inter-relationships between PV and CRF, PA, SES and NAPLAN results. The two extreme quartile score groups from the measures of PV, PA and CRF were examined in relation to NAPLAN scores. PV scores arising from testing with a small visual angle and SES were found to be significantly associated with overall academic scores, and with the Numeracy, Reading, and Writing components of academic performance. Female gender was significantly associated with Writing score, and male with Numeracy score. Being less influenced by the background tilted frame, and therefore having visual field independence (FI), was associated with significantly higher academic scores, with the largest effect in Numeracy scores (effect size, *d* = 0.82) and also associated with higher CRF and PA levels. FI was positively associated with all the academic modules examined, and most strongly with Numeracy test results, suggesting that FI provides an indicator of STEM ability. These findings suggest that further longitudinal research into strategies designed to enhance visual FI deserve consideration, with a focus on specialized PA programs for pre-pubescent children. It is possible that small visual angle spatial tasks during PA may stimulate neural networks involved in numerical cognition.

## Introduction

Enhanced levels of physical activity (PA) and cardio-respiratory fitness (CRF) in children are proposed to be positively associated with health, with wellness (Smith et al., 2014; Poitras et al., 2016) and also with academic performance (for a review, see Tomporowski et al., 2015; Ruiz-Ariza et al., 2017). While little controversy exists regarding the positive association of elevated levels of CRF and PA with enhanced child health and wellness outcomes (Smith et al., 2014; Poitras et al., 2016), some researchers have queried the extent to which PA and CRF are directly associated with academic performance, and have suggested the possible involvement of other factors (Pesce, 2012; Diamond and Ling, 2016). Diamond and Ling (2016) argue that PA undertaken with a focus on co-activating elements engaged in cognition provides the best possible combination of activities to enhance academic performance and cognitive function in children. Pesce (2012) has also suggested that while a significant portion of previous research has focused primarily on relationships between quantitative elements of PA with academic and cognitive ability, qualitative factors have been relatively neglected. Quantitative modes of PA relate exclusively to the duration and intensity of physical activities, whilst qualitative modes include more complex tasks requiring greater involvement of cognitive networks, by stimulating perceptual mechanisms and executive control pathways (Pesce, 2012; Tomporowski et al., 2015).

Spatial ability includes both a perceptual component and a qualitative factor associated with PA (Pesce, 2012). Spatial ability relates to the organization of sensory experiences in perception of physical spaces, incorporating embedded objects and the perceiver. Understanding the association between spatial perception and PA is important for pedagogical reasons, as evidence suggests that spatial abilities strongly predict analytical aptitude, involvement and performance in Science, Technology, Engineering and Maths (STEM) education (Shea et al., 2001; Wai et al., 2009; Ganley et al., 2014). Recently, a working paper by the OECD suggested that spatial ability and STEM learning are associated, and that spatial abilities are malleable (Newcombe, 2017).

An individual’s ability to estimate the direction of gravitational vertical, known as subjective visual vertical (SVV) is an important spatial ability when selecting a frame of reference to be used in the organization of upright orientation and postural alignment, and it requires both sensorimotor and cognitive participation (Isableu et al., 2010; Agathos et al., 2015). To successfully engage in any PA, an individual must select a suitable sensory frame of reference for spatial orientation so as to functionally align their body axis with primary vertical and horizontal axes, by means of information collected from vestibular and somato-sensory receptors and/or from visual examination of the physical features of their environment (Isableu et al., 2010; Agathos et al., 2015).

VV predictions begin with the engagement of the vestibular system providing a head centered frame of reference for upright orientation and alignment (Tarnutzer et al., 2009). Specifically, the saccule otolith is the primary sensory apparatus for detecting linear accelerations and head tilts in the vertical plane, with the signal disambiguated by integration of input from the posterior semi-circular canal detecting angular acceleration in the frontal plane. These integrated signals are then combined with roll plane proprioceptive inputs from the cervical spine enabling the head to be isolated as the segment exploited to form the SVV-based head stabilized in space strategy (Assaiante and Amblard, 1993; Tarnutzer et al., 2009; Schuler et al., 2010; Clemens et al., 2011; Medendorp and Selen, 2017). The central nervous system, using probabilistic methods, integrates and re-weights these multi-sensory inputs from multiple sources to formulate the most reliable spatial frame of reference and thereby align the body to gravitational vertical (Carver et al., 2006). Recent theory also holds that the central nervous system can internally construct a prediction of the direction of gravitational vertical, based on past experiences, when sensory information is ambiguous, unreliable or inaccessible (Mergner et al., 2003; Carver et al., 2006; MacNeilage et al., 2007; Barra et al., 2010).

Many spatial tasks require the central nervous system to estimate physical spatial quantities (kinematic or dynamic) and this is the basis for the theory of dimensional magnitudes as a foundation for numeracy (Walsh, 2003). For example, dimensional magnitudes include displacement and velocity, directions and orientation in three dimensional Euclidean space with force, mass or inertia related to a target or to the body. These physical properties are bound to dimensions and quantities and are embodied in their cognitive relationships (Koziol et al., 2012). Further, these physical properties are estimated relative to a spatial frame of reference, with the foundational one being SVV. Therefore to estimate distance, dimensions or other physical properties immersed in the spatial world, SVV predictions underlie their cognitive representation. This may then serve as a scaffold for spatial cognition and its association with STEM subjects. Support for this proposition also comes from Verdine et al. (2017), stating that the foundation of mental models representing numbers and magnitudes may be in spatial representations.

SVV is typically measured using the rod and frame test (Witkin and Asch, 1948; Oltman, 1968; Isableu et al., 2010). In all versions of the rod and frame test, an individual is positioned in a darkened environment and exposed to a tilted illuminated frame encasing a movable, illuminated rod, tilted from the vertical. Individuals are tasked with aligning the rod to gravitational vertical, with error in doing so thus providing a measure of SVV.

Two primary strategies exist in the formulation of gravitational vertical estimations in humans. First, the tilted frame may strongly affect ability to align the rod to vertical. Those affected typically use a visually dominant strategy that is heavily reliant on sensory inputs from peripheral vision (the frame), leading to biased estimation of vertical (Isableu et al., 2010) and these individuals are classified as visually field dependent. Evidence suggests that field dependent participants experience difficulties in up-weighting internal postural signals (vestibular and somatosensory) for obtaining more accurate perceptual constructs (Isableu et al., 2010; Agathos et al., 2015). Further, Agathos et al. (2015), provide evidence that field independent participants possess higher selective attentional control, more stabilized eye movements and greater inhibitory capabilities than field dependent subjects, with significant advantages in academic tasks, particularly STEM performance.

Secondly, individuals whose perception of vertical (PV) is more independent of the visual frame are said to use a field independent strategy. Field independent individuals most often employ a flexibly arranged and re-weighted integration of vestibular cues and somatosensory inputs with vision for PV (Isableu et al., 2010). This strategy provides a task-specific prediction of gravitation vertical which reliably provides lower error in the estimate of vertical than does the field dependent strategy, and applies even when confronted by ambiguous, conflicting or challenging visual environments (Kent-Davis and Cochran, 1989; Isableu et al., 2010). Finally, with extensive experience of sensory signals related to vertical alignment, predictive mechanisms are refined leading to an internal prediction of the direction of gravitational vertical (Lopez et al., 2011) that can act as a critical arbiter in sensorily deprived environments (Mergner et al., 2003; Barra et al., 2010).

Early researchers into the field independent and field dependent phenomenon suggested the rod and frame test and a test of spatial cognition – the Embedded Figures Test – to examine similar properties of spatial ability, and these were at first used for field dependent and field independent classification. However upon further review, correlation analysis found that, whilst both measures of field independent and field dependent share similar characteristics, they do not measure the same thing. For example from over 300 studies, Arbuthnot (1972) found the correlation between the two measures to be moderate to strong but indicating that they were not inter-changeable. It is a widely held view that the Embedded Figures Test is a more effective test of spatial cognition in that it reflects a child’s ability to perform numeracy tasks. Classification of field dependent and field independent should stipulate the type of measure used in assessment (Arbuthnot, 1972).

Previous longitudinal research examining field dependence and independence in young children through to adolescence indicates that there is progress from being relatively strongly field dependent to being relatively more field independent by adulthood; with boys more field independent than girls (Witkin et al., 1967; Bagust et al., 2013). At an individual level, a child from about 7 years can be classified as relatively field independent or field dependent compared to a cohort of similarly aged children. As the child in this group ages, they will generally become more field independent but their ranking relative to other subjects in this group will not significantly change (Witkin et al., 1967).

Both early research and more contemporary studies provide evidence of high inter-individual variability in the accuracy of predicting vertical using the rod and frame test, whilst within the individual there is a stable preference for the style or mode of vertical perception, leading to self-consistency in performance on a number of spatial tasks (Witkin, 1959; Isableu et al., 2010). This prompted early researchers to identify PV as a perceptual style, whereby individuals are ranked according to their high or low error rate on performance of tasks measuring vertical perception, and then grouped as either field dependent or field independent (Witkin, 1959). Conventionally, a group of individuals performing the rod and frame test is divided into top and bottom quartiles, based on their rod and frame test results (Isableu et al., 1997). The low perceptual error quartile is classified as field independent and the highest perceptual error quartile classified as field dependent.

Field independent and field dependent individuals appear to analyze information differently, using different cognitive strategies and have different levels of cognitive neural complexity in problem solving, with field independent using an analytic strategy by breaking down the complex structure of a stimulus, and field dependent employing a more holistic style (Witkin and Goodenough, 1981; Davis and Cochran, 1982; Jia et al., 2014; Agathos et al., 2015). Field independent individuals are also described as having an articulated body concept and percept, in which they view the available sensory array as discrete and have the ability to cognitively restructure available information to formulate more accurate perceptual predictions (Witkin, 1959; Isableu et al., 2010; Agathos et al., 2015).

Expanding on the relationship between cognitive functions and rod and frame test performance in measuring SVV, research has consistently revealed differences in general learning and memory between field dependent and field independent individuals, as determined by the rod and frame test (Gough and Olton, 1972; Goodenough, 1976; Blowers and O’Connor, 1978; Shinar et al., 1978; Berger and Goldberger, 1979; Amador-Campos and Kirchner-Nebot, 1999). Evidence from the Wechsler Intelligence Scale for Children supports the view that field independent children perform with greater effectiveness in tests of intelligence (Goodenough and Karp, 1961; Dreyer et al., 1971).

Whilst evidence supports an association between rod and frame test performance and cognition, controversy still exists regarding the association with academic performance in pre-adolescent children (**Table 1**). Support for the argument that the rod and frame test is related to elements of academic performance comes from Kagan and Zahn (1975), Kagan et al. (1977) and Canavan (1969), all of whom used a mechanical version of the RFT called the “Man in the Frame” rod and frame test, with a smaller visual angle of 22 degrees. Alternatively, several studies using the traditional mechanical rod and frame test with a larger visual angle of 28 degrees (Oltman, 1968) have found no significant relationship between rod and frame test results and measures of academic performance (Buriel, 1978; Allan et al., 1982; Wong, 1982; Tinajero and Páramo, 1997).

**Table 1:** Summary of childhood studies into the association of between rod and frame tests measuring subjective visual vertical using either a small or large visual angle with academic performance.

Study methodology						Academic association		

**Study**	**Age**	**N**	**Test**	**Visual angle**	**Research**	**Results association with rod and frame test**	**Numeracy**	**Reading English**
Kagan et al., 1977	10–12	230	MF	22° small	Correlation	Maths and English moderate to strong correlation	✓	✓
Kagan and Zahn, 1975	8–12	134	MF	22° Small	Correlation and multiple regression	Sig correlation maths (β = −0.22) and English (β = −0.19)	✓	✓
Canavan, 1969	10–12	1167	MF	22° small	Correlation	Maths and English moderate to strong correlation	✓	✓
Buriel, 1978	7–10	80	Hybrid large visual angle, MF RFT design	28° large	Correlation ANOVA	No relationship maths or English	×	×
Wong, 1982	10–11	90	PRFT	28° large	Correlation regression	No relationship maths, English or language	×	×
Tinajero and Páramo, 1997	13–16	408	PRFT	28° large	MANOVA	No significant source of variance languages, science, maths, English and overall	×	×

*Methodology: man in the frame (MF) RFT with visual angle 22°, portable rod and frame test (PRFT) with visual angle 28° Hybrid – portable rod and frame test with the man in the frame visual task.*

The reported associations between SVV, PA and motor coordination are unambiguous. Field independent compared to field dependent individuals have higher levels of PA and sports participation, and are seen as possessing greater sports potential (Liu and Chepyator-Thomson, 2008, 2009), more advanced motor comptency and motor coordination (Meek and Skubic, 1971; Golomer et al., 1999) and greater ability to learn novel motor tasks (Hodgson et al., 2010). Finally, more athletes are field independent than non-athletes (Brady, 1995). Most of the studies reviewed examined SVV using the mechanical rod and frame test with a visual angle of 28 degrees (Oltman, 1968). The rod and frame test appears to be able to distinguish between athletes and non-athletes due to the higher engagement of vestibular and proprioceptive sensors (Raviv and Nabel, 1990; Liu and Chepyator-Thomson, 2009).

**FIGURE 1:**
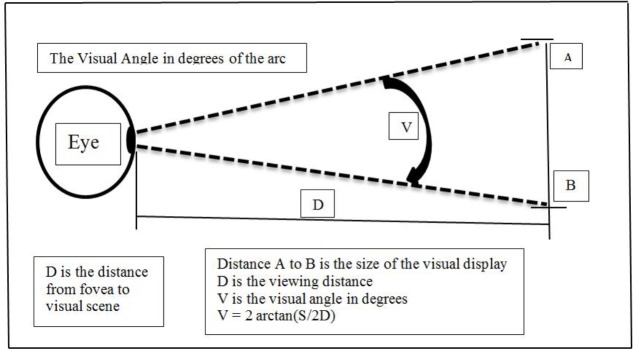
Determinants of the visual angle.

The visual angle projected onto the eye during the rod and frame test is an important factor in SVV performance. The visual angle is produced by two straight lines drawn from the peripheral points of a seen object to the fovea of the eye and is normally stated in degrees of arc (**Figure 1**), and visual angles are an important discriminating characteristic between small and large field of view environments in spatial perception (Wang et al., 2014).

Large-scale field-of-view environments naturally produce large visual (retinal) angles. When the participant is an active part of the environment the PV incorporates the involvement of proprioceptive and vestibular input, causing visual-vestibular interactions generated by the head, body and eye movements that are made in order to take in the full visual array. Research suggests that large visual angles promote perceptions related to environmental interactions, such as movement and navigation, and may be more closely associated with PA measures (Quaiser-Pohl et al., 2004). On the other hand, perceptions formed by small scene environments characteristically employ small visual angles with reduced eye, head or body movements and are more reliant on foveal-based ventral visual streams. Quaiser-Pohl et al. (2004) provide evidence that small visual angle RFT scores have a moderate to strong correlation with other small-scale environment spatial cognitive tasks, the Water-Level-Task and a Mental-Rotations-Test, in 10–12 years old children, while large scale environments with large visual angles were uncorrelated with the small visual angle RFT. Quaiser-Pohl et al. (2004) suggested that it is the small-scale visual environments that tap directly into the spatial reasoning mechanisms associated with STEM subjects.

Finally, SES was included as it has been found to be a strong predictor of academic performance (Mezzacappa, 2004; Sirin, 2005) and CRF (Duncan et al., 2002) and has previously been associated with performance with the rod and frame test (Maceachron and Gruenfeld, 1978). Therefore, including SES as a benchmarking measure enables the assessment of the relative importance of different associations.

The current study examined the cross-sectional relationships between PA, CRF, SES, academic performance and a Computerised Rod And Frame Test (CRAFT) in 10-year-old children. This study is the first to examine PV using a small visual angle CRAFT in association with both academic and physical health-related measures in a large cohort of 10 years old children.

The primary hypothesis was that measurement of SVV using a small visual angle would be significantly associated with measures of academic performance, with the greatest association arising in numeracy scores and would generate significant effects when comparing field dependent with field independent individuals. It was further hypothesized that the SVV results would have significantly smaller associations with PA-related measures, in a manner different from findings in previous studies using a large visual angle task. Finally, it was proposed that grouping participants into the highest and lowest PA and CRF quartiles would show that children who exhibit higher CRF and PA levels have a significant advantage in areas of academic performance and support the role of enhanced PA levels as a moderating effect on academic performance.

## Materials and Methods

### Participants

The data were collected as part of the Lifestyle of our Kids (LOOK) that included 853 (418 females) children with mean age 11.3 years [(SD 0.3) years] in a longitudinal study that involved 29 elementary (primary) schools in Canberra in the Australian Capital Territory (Telford et al., 2009). From the total LOOK sample there were 341 (162 females) children who performed all the tests required for the current study. The numbers of children in the regression analyses on academic score dependent variables were; Overall Academic Score (*n* = 341), Numeracy (*n* = 345), Reading (*n* = 345) and Writing (*n* = 346). The difference in the numbers of children in this study compared to the total involved in the research was primarily related to access to children on testing days. Elements of testing were conducted at different times, some at school and some at a hospital location, and if children did not attend school on the relevant testing days, their data was not collected. The measure of SES used in study was obtained from a parent questionnaire on the level of education achieved, however not all parents returned this questionnaire. Further, some children did not sit the Australian National Assessment Program Literacy and Numeracy (NAPLAN) exam due to parental choice. For the PA measure, the children were encouraged to wear the pedometer each week, however not all did so.

Academic tests were conducted in the same year as with the other measures, except for the CRAFT with data collected 4 months before the academic tests. The relative uniformity of the schools in the study reflected the fact that all schools were part of a local public education system, receiving similar funding. This study was approved by the Australian Capital Territory Health and Community Care Human Research Ethics Committee. Participation by the children was voluntary and informed consent for involvement was received from parents or guardians.

### Measures

#### Perception of Vertical Measurement

With the CRAFT, a 430 mm flat screen monitor presented an image of a tilted illuminated frame enclosing a tilted illuminated rod. The square illuminated frame was 185 mm wide and surrounded by a blackened cylinder 330 mm diameter and 500 mm long (Haynes et al., 2008). The viewing tube was set 30 mm from the computer screen. The computer screen, viewing tube and child were all covered by a dark cloth eliminating any external light source. When tested, the child was seated with the viewing tube at eye level and their chin placed on a foam rest within the entrance of the viewing tube, and to limit proprioceptive and vestibular inputs during the trials the child was requested to remain still. The visual angle of twenty degrees was calculated from the distance of the chin rest on the viewing tube to the square frame appearing on the computer screen. Designing the CRAFT with a small visual angle and reducing head motion enabled measurement of a child’s prediction of gravitational vertical with reduced visual-vestibular interactions and proprioceptive inputs.

Children were asked to imagine that the rod was a rocket ship aimed to shoot straight up to an imaginary moon. The children were told that in the event the rocket ship was not pointing straight up, then it would crash. The child was given two practice trials to “launch the rocket ship to the moon.” If performance was poor, the child was shown the error and instructed on the correct alignment.

**FIGURE 2:**
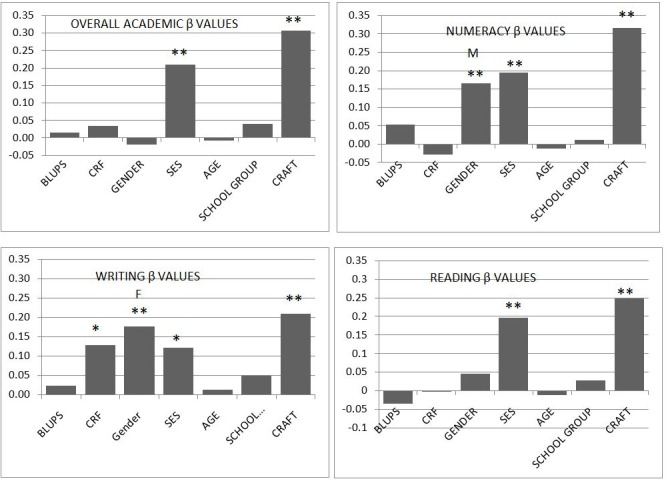
Beta weights from the hierarchical regressions. Dependent variables are Overall academic scores. Numeracy, Reading, and Writing. Independent variables are Physical Activity (PA), Cardio-respiratory Fitness (CRF). Socio-Economic Status (SES) and Computer Rod And Frame Test (CRAFT). CRAFT produced negative beta values because low error scores are associated with higher scores on academic variables, however for comparison with the other independent variables, the CRAFT beta values are shown here as positive. Gender beta values are shown as positive for the better performing gender – MALE (M) and FEMALE (F). No changes were made to the signs of the beta values for PA, CRF, SES. Significance: ^∗^*P* ≤ 0.05, ^∗∗^*P* ≤ 0.01.

The test consisted of ten trials presented in random order, consisting of five frames tilted clockwise and five frames tilted anti-clockwise at eighteen degrees to vertical. The rod was positioned either twenty degrees positive or negative to vertical with the frame tilted eighteen degrees clockwise or counter-clockwise (**Figure 2**). The child could move the rod using a handheld mouse and when satisfied with the “rocket ship” alignment, pressed the space bar to record their response, thereby giving an angle from the vertical, and were then automatically moved onto the next trial. The mean of the ten absolute (unsigned) errors for each subject was calculated. The reliability of the mechanical rod and frame test has been previously found to be acceptable (Witkin et al., 1967).

#### Socio-Economic Status Measurement

The measure of SES employed was obtained from a questionaire enquiring about the level of educational attainement by either of the parents. Score of one indicated year 10 level, score two was high school level of education and score three was tertiary qualification. Because SES has been found to be a strong predictor of academic performance (Sirin, 2005), knowing SES is critical in any study of academic performance differences.

#### Physical Activity and Physical Fitness Measurements

Cardio-respiratory fitness was assessed by a twenty-meter multistage run (Tomkinson et al., 2003) using methods described by Telford et al. (2009). Participants ran between two lines, 20 m apart, while keeping pace with a loud beeping sound arising from a sound amplifier. The measure of CRF was the number of stages reached in the multistage run.

Physical activity was measured by AT pedometers (New-Lifestyles, Lee’s Summit, MO, United States) considered to be sufficiently valid and reliable (Beets et al., 2005) as described by Telford et al. (2009). PA was measured by pedometer use for seven consecutive days by every child, conducted as described in Telford et al. (2009). A PA index was formulated engaging the Best Linear Unbiased Predictor (BLUPS) (Robinson, 1991).

#### Academic Performance Measurement

Academic performance was measured by the NAPLAN tests conducted in year five^1^. The Australian Department of Education is responsible for all testing and scaling of results collected from all Australian primary school children. Numeracy, Reading, and Writing scores were collected from the NAPLAN results for the current project. Persuasive and narrative writing skills were examined in the Writing task. Literacy proficiency is tested by reading tasks, and focuses on the reading and comprehension of written English. The Year five reading text included biographies, autobiographies and persuasive passages. Several competencies were examined in the Year five NAPLAN numeracy test, including algebra, quantities, patterns and measurements and space concepts.

### Statistical Analysis

Descriptive statistics were obtained first. Bivariate correlations between the procedures were calculated as a validity check, to determine if the individual variables within a class had significant relationships. Correlation analysis used the maximum number of participants available so as to get the best estimate of descriptive statistics. Effect size for bivariate correlations using Pearson’s coefficient of correlation was determined using the classification provided by Cohen (1992), with a small effect at 0.1, medium at 0.3 and large at 0.5.

To examine the contribution of SVV in the explanation of academic performance in ten year old children, a hierarchical multiple regression analysis was performed. A series of three-step hierarchical linear regressions were undertaken on dependent variables – Year five NAPLAN results in Reading, Writing, Numeracy and overall academic scores. Independent predictor variables were: CRF, PA, CRAFT, and SES. As well, sex (male, female) was included as a dichotomous variable in the hierarchical linear regression model. All statistical assumptions were met in the analyses. In step one, independent variables PA and CRF were regressed. Sociocultural factors – SES and the dichotomous gender variable were included in step two. The third step involved including the measure of SVV measured with the CRAFT. In order to adjust significance level for multiple comparisons, the Bonferroni correction was employed and the alpha level was set at 0.01.

The upper and lower quartiles were determined for all independent variables. The two groups were defined as “first quartile” (children who achieve the highest competency in a task) and “fourth quartile” (children who have the lowest competency in a task).

Students in the first quartile (low CRAFT error scores indicating an ability to accurately assess gravitational vertical) were classified as field independent; and fourth quartile participants (high error in predicting vertical) and were classified as field dependent. Quartile splits for PA and CRF were also applied to the academic variables. To assess the influence of classification of individuals as field independent or field dependent, independent groups *t*-tests were applied to the quartiles produced from the CRAFT, first with dependent variables Numeracy, Reading, Writing and overall academic performance, as well as PA CRF, and SES. Independent-groups *t*-tests were also performed on the quartile splits on PA and CRF scores with dependent measures; Reading, Writing, Numeracy and overall academic score, and with CRAFT performance. Means and standard deviations were calculated for Reading, Writing, Numeracy and overall academic scores to determine overall academic performance in the different groups. Using the quartile mean scores and standard deviation, effect size was calculated using Cohen’s d, the standardized mean difference, with a small effect designated as 0.2, medium as 0.5 and large as 0.8 (Cohen, 1992). Statistical analyses were performed using SPSS version 21 with statistical significance set at 0.05.

## Results

**Table 2** provides descriptive statistics. Bivariate correlations between the three measures of academic performance from the national examination scheme (reading, writing, and numeracy) and summed academic scores, together with the CRAFT, CRF, PA, and SES results are presented in **Table 3**.

**Table 2:** Descriptive statistics.

Independent variable	*N*	Min	Max	Mean	St. Dev
CRAFT	541	0.8	55	5.6	5.7
Overall academic scores	705	202.33	716.7	489.7	62.8
Numeracy	714	307.00	771	484.8	71.5
Writing	716	89.00	692	483.5	71.7
Reading	715	98.00	754	499.5	77.9
SES	593	1	3	2.49	0.72
PA	853	64.99	133.7	100.1	11.1
CRF	534	1.30	11.5	5.34	1.9
Valid (listwise)	341				

*Sample means, standard deviation (St. Dev) minimum (min) and maximum (max). Computerised Rod And Frame Test (CRAFT), Socio-Economic Status (SES), Cardio-Respiratory Fitness (CRF), and Physical Activity (PA). SES score represents the highest educational attainment by a parent with one representing lowest level of education with three representing a university degree or similar. PA score represent an index of PA with a low score indicating low levels of PA and high scores representing high levels of PA CRF represented by a shuttle run score with the number of stages completed.*

**Table 3:** Bivariate correlation coefficient matrix.

		1	2	3	4	5	6	7	8
1. CRAFT	*r*	1	−0.31^∗∗^	−0.345^∗∗^	−0.2^∗∗^	−0.243^∗∗^	−0.111^∗^	−0.078	−0.056
	Sig.		0.001	0.001	0.001	0.001	0.021	0.07	0.239
	N	541	507	513	514	514	429	541	442
2. OAS	r		1	0.852^∗∗^	0.806^∗∗^	0.896^∗∗^	0.274^∗∗^	0.074	0.116^∗∗^
	Sig.			0.001	0.001	0.001	0.001	0.051	0.009
	N		705	705	705	705	526	705	503
3. NUMERACY	r			1	0.493^∗∗^	0.691^∗∗^	0.254^∗∗^	0.146^∗∗^	0.12^∗∗^
	Sig.				0.001	0.001	0.001	0.001	0.007
	N			714	708	709	532	714	510
4. WRITING	r				1	0.582^∗∗^	0.209^∗∗^	0.051	0.146^∗∗^
	Sig.					0.001	0.001	0.173	0.001
	N				716	711	532	716	512
5. READING	r					1	0.24^∗∗^	−0.007	0.034
	Sig.						0.001	0.846	0.449
	N					715	533	715	510
6. SES	r						1	−0.01	0.032
	Sig.							0.806	0.52
	N						593	593	414
7. PA	r							1	0.487^∗∗^
	Sig.								0.001
	N							853	534
8. CRF	r								1
	Sig.								
	N								534

*Significance and number of participants for 10 years old children for the Rod and Frame Test (RFT), Cardio Respiratory Fitness (CRF), Physical Activity (PA), Socio-Economic Status (SES) and NAPLAN data from summed academic scores and individual results in Reading, Writing, and Numeracy. ^∗^Correlation is significant at the 0.05 level (2-tailed). ^∗∗^Correlation is significant at the 0.001 level (2-tailed).*

Small to medium-sized, statistically significant correlations were observed between the CRAFT and individual measures of academic performance, measured by the NAPLAN tests in ten year old school children for reading (*r* = −0.24, *p* < 0.001), writing (*r* = −0.2, *p* < 0.001), numeracy (*r* = −0.35, *p* < 0.001) and summed academic scores (*r* = −0.31, *p* < 0.001). Small to medium sized correlations were found between SES and academic performance in reading (*r* = 0.24, *p* < 0.001), writing (*r* = 0.21, *p* < 0.001), numeracy (*r* = 0.25, *p* < 0.001) and overall academic scores (*r* = 0.27, *p* < 0.001).

Cardio-respiratory fitness association with numeracy was significant and classified as small (*r* = 0.12, *p* = 0.007), as it was with Overall Academic Scores (*r* = 0.12, *p* = 0.009), and with writing having a small to medium effect size (*r* = 0.15, *p* < 0.001). No association was discovered between CRF and reading. PA had a small to medium, significant relationship with numeracy (*r* = 0.15, *p* < 0.001) only.

Socio-economic status had no significant correlation with CRF or PA. SES produce a small significant association with CRAFT (*r* = −0.11, *p* = 0.021). Moderate and significant correlations were observed between PA and CRF (*r* = 0.49, *p* < 0.001). Moderate to large and significant correlations were found between numeracy and reading (*r* = 0.69, *p* < 0.001), numeracy and writing (*r* = 0.49, *p* < 0.001) and reading and writing (*r* = 0.58, *p* < 0.001). PA and CRF correlation scores with the CRAFT were not significant (all *p* > 0.1).

Hierarchical linear regression analysis is exhibited in **Table 4**. Beta values were tabulated from the regression analysis in individual column charts (**Figure 1**) for dependent variables; Overall academic scores, numeracy, reading and writing with independent variables being PA, CRF, SES, and CRAFT score. In relation to overall academic scores, hierarchical linear regression analysis results from step one revealed no significant relationships with PA and CRF variables. Step two was significant (*R*^2^ = 0.09) and provides evidence of a significant association with overall academic scores arising from SES. No significant association found from the gender variable. Step three including only the CRAFT variable with scores tending to be higher (greater field dependent) for poorer academic scores, giving a negative B-weight, and PV variable alone accounted for 8.4% of the summed overall academic scores variance. CRAFT measurement performance (β = −0.3; *t* = −5.85, *p* < 0.001) had significantly higher beta values than SES measures (β = 0.26; *t* = 5.22, *p* < 0.001). The model accounted for 16.8% of the variance.

**Table 4:** Hierarchical regression models predicting academic performance in primary schoolchildren.

Model	B	Std. Error	β	*t*	Sig.	Adj R^2^	R^2^ Change	Sig F Change
**Overall academic scores (*N* = 341)**								
(Constant)	476.458	26.841		17.751	0.001			
Step 1_PA_	0.114	0.304	0.024	0.375	0.708			
CRF	2.253	1.960	0.073	1.150	0.251			
						0.002	0.008	0.275
(Constant)	417.747	27.626		15.122	0.001			
Step 2_PA_	0.026	0.299	0.005	0.085	0.932			
CRF	1.711	1.895	0.055	0.903	0.367			
SES	24.376	4.263	0.297	5.718	0.001			
GENDER	6.481	6.547	0.055	0.99	0.323			
						0.086	0.089	0.001
(Constant)	457.460	27.214		16.810	0.001			
Step 3_PA_	0.000	0.285	0.001	0.001	1.000			
CRF	1.253	1.810	0.04	0.692	0.489			
SES	21.381	4.099	0.26	5.216	0.001			
GENDER	0.64	6.325	0.005	0.101	0.92			
CRAFT	−3.335	0.57	−0.297	−5.851	0.001			
						0.168	0.084	0.001
**Numeracy (*N* = 345)**								
(Constant)	419.180	32.537		12.883	0.001			
Step 1_PA_	0.693	0.369	0.118	1.879	0.061			
CRF	0.822	2.382	0.022	0.345	0.73			
						0.011	0.017	0.054
(Constant)	349.968	33.091		10.576	0.001			
Step 2_PA_	0.3	0.358	0.051	0.839	0.402			
CRF	−0.953	2.27	−0.025	−0.42	0.675			
SES	25.939	5.111	0.257	5.075	0.001			
GENDER (M)	36.167	7.853	0.250	4.606	0.001			
						0.121	0.114	0.001
(Constant)	395.386	32.548		12.148	0.001			
Step 3_PA_	0.29	0.341	0.049	0.85	0.396			
CRF	−1.538	2.170	−0.04	−0.709	0.479			
SES	22.183	4.923	0.22	4.506	0.001			
GENDER (M)	29.544	7.584	0.205	3.895	0.001			
CRAFT	−3.953	0.679	−0.288	−5.821	0.001			
						0.199	0.079	0.001
**Reading (*N* = 345)**								
(Constant)	532.417	33.362		15.959	0.001			
Step 1_PA_	−0.31	0.378	−0.052	−0.82	0.413			
CRF	1.674	2.425	0.043	0.69	0.49			
						−0.004	0.002	0.68
(Constant)	463.278	34.585		13.396	0.001			
Step 2_PA_	−0.318	0.373	−0.053	−0.853	0.394			
CRF	1.359	2.358	0.035	0.577	0.565			
SES	28.854	5.295	0.284	5.449	0.001			
GENDER	−0.335	8.153	−0.002	−0.041	0.967			
						0.062	0.059	0.001
(Constant)	503.095	34.69		14.503	0.001			
Step 3_PA_	−0.343	0.362	−0.057	−0.947	0.344			
CRF	0.87	2.293	0.023	0.379	0.705			
SES	25.844	5.185	0.254	4.984	0.001			
GENDER	−6.11	8.019	−0.042	−0.762	0.447			
CRAFT	−3.349	0.726	−0.239	−4.611	0.001			
						0.101	0.041	0.001
**Writing (*N* = 346)**								
(Constant)	477.854	29.519		16.188	0.001			
Step 1_PA_	−0.051	0.335	−0.01	−0.153	0.879			
CRF	4.203	2.149	0.122	1.956	0.051			
						0.008	0.014	0.091
(Constant)	434.516	30.994		14.019	0.001			
Step 2_PA_	0.107	0.334	0.02	0.319	0.75			
CRF	4.642	2.112	0.135	2.198	0.029			
SES	19.219	4.752	0.211	4.044	0.001			
GENDER (F)	−15.500	7.326	−0.119	−2.116	0.035			
						0.072	0.08	0.001
(Constant)	465.396	31.316		14.862	0.001			
STEP 3_PA_	0.09	0.327	0.017	0.276	0.783			
CRF	4.251	2.070	0.124	2.054	0.041			
SES	16.865	4.69	0.185	3.596	0.001			
GENDER (F)	−20.030	7.261	−0.153	−2.759	0.006			
CRAFT	−2.618	0.657	−0.209	−3.982	0.001			
						0.124	0.054	0.001

*Cardio-Respiratory Fitness (CRF), Physical Activity (PA), Percent of Body Fat (% BF), Socio-Economic Status (SES) and Computerised Rod And Frame Test (CRAFT). Bonferroni correction for multiple comparisons set significance at *p* < 0.01.*

In explanation of Numeracy, hierarchical multiple regressions revealed that from step one, neither CRF nor PA contributed significantly to the variance of the model. Introducing step two explains an additional 11.4% of variance with SES and male gender both significant. Step three provides evidence of 19.9% of variance in the model (adj *R*^2^ = 0.2) with the CRAFT variable explaining 7.9% of the variance of the model. Significant beta values in step three were PV score (CRAFT) (β = −0.288; *t* = −5.821, *p* < 0.001), SES (β = 0.22; *t* = 4.51, *p* < 0.001) and sex (male) (β = 0.21; *t* = 3.895, *p* < 0.001).

The results of step one, when examining the Reading dependent variable, reveal PA and CRF variables produce no significant association. In step two, SES and gender dichotomous variable were entered, with only SES explaining variance in the model (*R*^2^ = 0.59). When all five variables were included in the step three model, only SES (β = 0.254; 4.984; *p* = < 0.001) and SVV (β = −0.239; *t* = −4.611; *p* < 0.001) variables were significant. SVV variable measured by the CRAFT explained 4.1% of the variance. Together the six independent variables accounted for 10.1% of the variance in Reading scores.

To examine the unique contribution of SVV in the explanation of writing skill in eleven year old children, a three step hierarchical multiple regression analysis was performed. Step one examining PA and CRF variables provided evidence of a significant small association arising in CRF. 1.4% of the variance arising in the model was explained in step one. By step two a further significant 8% of the variance is explained in the model with SES and gender (female) significant in their contribution. Step three, including the five variables in the model, provides 12.4% of the variance explained by the model, with SVV explaining 5.4% of the model. The three independent variables significantly related to the results in this model were SES (β = 0.19; *t* = 3.6; *p* < 0.001), SVV (β = −0.21; *t* = −3.98; *p* < 0.001) and gender (female) (β = −0.15; *t* = −2.76; *p* = 0.006).

To compare the effects of classification regarding a field independent or field dependent perceptual style, with the quartiles produced from the CRAFT, *t*-tests were applied to examine overall academic scores, numeracy, reading and writing (**Table 5**). Numbers of participants classified as field dependent ranged from 130 to 140 participants, whilst field independent participants also ranged from 130 to 140 participants; depending on the variable of interest. Significant differences between the first (field independent) and fourth (field dependent) quartiles obtained from independent-groups *t*-tests for the CRAFT were observed in Numeracy [*d* = 0.82; (*t*_265_) = 6.71, *p* < 0.001], Overall academic scores [*d* = 0.65; (*t*_263_) = 5.29, *p* < 0.001], Reading [*d* = 0.47; (*t*_264_) = 3.859, *p* < 0.001) and Writing [*d* = 0.36; (*t*_264_) = 2.960, *p* = 0.003] with the field independent group outperforming the field dependent group on each variable. PA was positively associated with field independent perceptual style [*d* = 0.4; (*t*_277_) = 3.342, *p* = 0.001] and CRF [*d* = 0.26; (*t*_215_) = 1.913, *p* = 0.057] There was no significant difference between field independent and field dependent individuals in SES.

**Table 5:** High and low competency quartile groups in rod and frame test, cardio-respiratory fitness and physical activity.

					Independent *t*-tests				
Independent Variable	Mean	SD	first Quartile	fourth Quartile	d	t	df	sig.	Mean Diff
**Computer Rod And Frame Test (Subjective visual Vertical)**									
Overall academic scores	490	(62.8)	508 (64.3)	468 (58.3)	0.65	5.294	263	0.001	39.6
Numeracy	485	(71.5)	508 (69)	452 (66.8)	0.82	6.711	265	0.001	55.8
Reading	499.5	(77.9)	517 (83)	480 (74.8)	0.47	3.859	264	0.001	37
Writing	483.5	(71.7)	497 (80)	470 (62.9)	0.36	2.960	264	0.003	26.1
Socio-economic status	2.49	(0.72)	2.56 (0.66)	2.42 (0.79)	0.19	1.425	219	0.156	0.14
Cardio-respiratory fitness	5.38	(1.9)	6.2 (2.1)	5.6 (2)	0.26	1.913	215	0.057	0.55
Physical activity	100.1	(11.1)	97.4 (12)	93 (10.8)	0.4	3.342	277	0.001	4.57
**Cardio-respiratory fitness**									
Overall academic scores	490	(62.8)	498 (60.5)	481 (68.2)	0.24	−1.988	262	0.048	15.8
Numeracy	485	(71.5)	494 (71.2)	473 (77.5)	0.29	−2.337	266	0.02	21.2
Reading	499.5	(77.9)	502 (78.3)	501 (73.7)	0.04	−0.376	267	0.707	3.6
Writing	483.5	(71.7)	495 (65.8)	472 (75.1)	0.33	−2.713	269	0.007	23.2
Physical activity	100.1	(11.1)	93 (10.7)	109 (11.3)	1.4	−12.230	283	0.001	−15.9
Subjective visual vertical	5.31	(4.65)	4.9 (5.1)	6.4 (6.5)	0.2	1.511	234	0.132	1.2
**Physical activity**									
Overall academic scores	490	(62.8)	496 (56.6)	480 (63)	0.28	−2.519	318	0.012	16.9
Numeracy	485	(71.5)	501 (66.7)	469 (68.8)	0.48	−4.306	322	0.001	32.4
Reading	500	(77.9)	499 (69.3)	495 (89)	0.05	−0.491	320	0.624	4.1
Writing	484	(71.7)	486 (61.9)	473 (80)	0.18	−1.619	324	0.106	12.8
Cardio-respiratory fitness	5.38	(1.9)	6.6 (1.9)	4.3 (1.5)	1.3	−10.920	267	0.001	−2.28
Subjective visual vertical	5.31	(4.65)	5.3 (5.9)	6.2 (5.4)	0.16	1.313	261	0.19	0.92

*Polar quartile groupings for Rod and Frame Test (RFT) denoting field independent and field dependent sub-groups with independent variables: Overall academic Scores, Numeracy, Reading, Writing, socio-economic status, physical activity and physical fitness. Cardio-respiratory Fitness and Physical Activity were also separated into quartiles based on high and low Physical Activity and Cardio-respiratory fitness with independent variables: Overall academic Scores, Numeracy, Reading and Writing. The Cohen’s d effect sizes for the CRAFT, CRF and PA quartile splits comparisons on Overall Academic Scores, Numeracy, Reading and Writing results. Cohen’s d defines small effect as 0.2, medium as 0.5, and large as 0.8.*

Fitter children measured by CRF were associated with enhanced Overall academic scores [*d* = 0.24; (*t*_262_) = −1.988, *p* = 0.05], Numeracy [*d* = 0.29; (*t*_266_) = −2.337, *p* = 0.02] and Writing [*d* = 0.33; (*t*_269_) = −2.71, *p* = 0.007]. More active children measured using pedometers were associated with Overall academic scores [*d* = 0.28; (*t*_318_) = −2.519, *p* = 0.012] and Numeracy [*d* = 0.48; (*t*_322_) = −4.306, *p* < 0.001].

## Discussion

In this study we examined the view that performance in the rod and frame test is primarily associated with PA measures (Liu and Chepyator-Thomson, 2008, 2009) and less associated with cognitive function as it has been claimed previously that the rod and frame test directly taps into body orientation mechanisms (Liu and Chepyator-Thomson, 2009). If the standard response in predicting the direction of gravitational vertical is for engagement of vestibular and proprioceptive mechanisms no matter the visual angle presented, then various PA variables should have strong and significant associations with the CRAFT measure.

The primary finding here indicates that the ability to accurately estimate gravitational vertical using this CRAFT methodology displayed significant relationships with academic performance, in particular numeracy ability, supporting predictions by Quaiser-Pohl et al. (2004). After controlling for PA and CRF, SES and gender; hierarchical regression analysis provided evidence of significant associations between all measures of academic success and SVV, most strikingly with numeracy. The strength of the relationship between academic success and accuracy in predicting vertical with the current methodology is emphasized by comparison to the effect of SES. CRAFT beta values were all larger than those for SES, except for reading. The association between SVV results and numeracy was the strongest of all the dependent variables measured, explaining 7.9% of the variance of the model. Numeracy had a less strong association, though significant, with SES and male gender. Being female was significantly associated with higher writing test scores.

The proposal that field independent children would have significantly better academic performance compared to that of field dependent children was also supported (**Figure 2**). All academic measures showed significant positive results for the field independent group, with moderate to large effect sizes in reading (*d* = 0.47) and overall academic scores (*d* = 0.65); and a small to moderate effect size in the writing task (*d* = 0.36). The most significant academic relationship associated with classification of field independent was numeracy, with a large effect size (*d* = 0.82), again providing possible evidence of an association with STEM cluster of cognitive abilities.

An important finding was the relatively small association between PA and CRF with CRAFT scores, where the level of association in both correlation and quartile comparisons was smaller than that found in previous studies. There were no significant correlations between PA and CRF with PV (all *r* > 0.1). In contrast, Liu and Chepyator-Thomson (2008) found Pearson correlations between PA levels and rod and frame test scores (using a 28 degree visual angle) of between −0.26 and −0.29 (*p* < 0.01) in 129 adolescents. In the current study, when children were separated into field dependent and field independent groups, a significant small to moderate effect size was produced from CRF, PA and percent body fat with the CRAFT. However, the effect size in the current study is significantly lower than what was found in other studies into PA levels using the field dependent – field independent classification (Liu and Chepyator-Thomson, 2009). In the Liu and Chepyator-Thomson (2009) study (using a portable rod and frame test with a larger visual angle of 28 degrees) these researchers found field independent adolescents to be significantly more physically active than field dependent participants and calculated the effect size to be large. The mean for field independent individuals’ PA levels was about twice that of FD individuals. In comparison, the present study field dependent - field independent classification provides evidence of a small to moderate positive effect size with PA, with the field independent group scoring 97.4 on the PA index and with field dependent participants averaging 93 in PA index, equating to a 3.5% difference in PA levels. The above findings may relate to the use of a small visual angle CRAFT. As in previous studies, SES was found to have small to moderate correlations with all academic variables (Sirin, 2005).

A possible explanation for the strong association observed here between general academic results and accuracy in predicting vertical, compared with relatively lower association for PA variables, is the measurement method used. The previous positive relationships noted between the “Man in the Frame” rod and frame test and academic variables (Canavan, 1969; Kagan and Zahn, 1975; Kagan et al., 1977) used a small visual angle of 22 degrees and the present study used a small visual angle of 20 degrees. Conversely, studies finding no relationship between the rod and frame test and academic performance have examined PV using the larger visual angle of 28 degrees (Buriel, 1978; Allan et al., 1982; Wong, 1982; Tinajero and Páramo, 1997). The relatively small visual angle employed in the present study means that the test can be classified as a small-scale environment assessment, producing limited visual-vestibular interactions.

Ebenholtz and Benzschawel (1977) first suggested retinal angle in the rod and frame test mediates the results of performance of the rod and frame effect. They further suggest that two related but separate mechanisms are engaged when using small and large visual angle rod and frame test tasks, in their “Dual Process Theory” (Ebenholtz and Glaser, 1982; Coren and Hoy, 1986). Ebenholtz (1977) first suggested that small and large visual angle performances in the rod and frame test are associated with the dual visual projection systems. On the one hand, a small visual angle is related to search fixation, identification, and grasping associated with use of the foveal visual system, whereas a large visual angle rod and frame test is more associated with peripheral visual systems designed for spatial orientation of self and objects in space and for navigation purposes. The Ebenholtz and Glaser (1982) study provides confirmation that large and small frame effects (related to small and large visual angles) are functionally dissimilar and associated with alternate but linked dorsal and ventral visual stream neural processing. Ebenholtz and Glaser (1982) suggest that the small visual angle RFT engages a foveal stimulated ventral visual stream sensory input, whilst a large visual angle produces a dorsal visual stream peripheral vision stimulus.

Streibel and Ebenholtz (1982) compared the performance of the rod and frame test using small and large visual angles and found a significant stimulus size (visual angle) effect, with the small visual angle rod and frame test (9 degrees) having a larger mean score than the large angle rod and frame test (41 degrees). Further, the Streibel and Ebenholtz (1982) findings support previous reports of poor correlations between perceptions arising from small and large scale environments (Spinelli et al., 1999; Quaiser-Pohl et al., 2004). In addition to this Quaiser-Pohl et al. (2004) concluded that a distinction should be made between children’s spatial cognition in small-scale and large-scale spatial environments, and that they measure definably different elements of spatial ability.

Supporting neurophysiological evidence is provided by a study by Lopez et al. (2011) using a rod and frame test with a small visual angle of 14 degrees, examining the sequence of neurologic activation patterns from electro-encephalograph event potentials during the rod and frame task. They found early activation in the right temporo-occipital cortex ventral stream; likely activating visual-visual mechanisms in the extrastriate cortex engaged at around 75 milliseconds and implicating attentional processes. Later, combined ventral stream and dorsal stream visual-vestibular zones are activated at around 260 milliseconds bilaterally in the temporal, occipital, and parietal areas. These results suggest engagement of temporal cortical zones are implicated in the frame effect for the purpose of maintaining internal estimates of vertical, while the parietal cortical areas are more involved with the control of posture, actions, and visuospatial processing.

We propose that the PV in the current study was most likely reliant on participants’ previous experiences of vertical alignment from vestibular and somato-sensory stimulation, then stored as an internal representation in temporal and parietal cortical zones. This requires significant cortical involvement to accurately predict gravitational vertical (Lopez et al., 2011). It is further proposed that children most capable of cognitive transformation of vestibular and somato-sensory inputs from previous experience into a stored internal representation of gravitational vertical have greater school academic success, particularly in numeracy. The observation of neural ventral visual stream engagement in a small angle rod and frame test (Lopez et al., 2011) may help explain the lack relationship found in the current study between CRAFT results with PA, CRF and percent of body mass measures. The small retinal angle produces cognitive responses more attuned to small scale environments and related cognitive processes (Quaiser-Pohl et al., 2004). It is possible enriched environmental experiences engaging small visual angle tasks may have developmental influences in forming this internal representation, together with inherited genetic advantage.

A second possible pathway modulating the association between CRAFT methodology examining SVV and academic performance is via attentional abilities, as outlined in previous studies (Blowers and O’Connor, 1978; Shinar et al., 1978; Berger and Goldberger, 1979; Agathos et al., 2015). The attentional theory may complement the proposition of small visual angle perception, in that the small field environment is proposed to stimulate attentional mechanisms more effectively (Quaiser-Pohl et al., 2004).

In this study, children’s performance on a test to determine visual vertical using a CRAFT provided evidence of a moderate to strong association with numeracy. Also, a field independent perceptual style was associated with a strong effect size in numeracy, compared to field dependent categorized children. These findings of relationships between subjective vertical perception and numeracy may be explained in a number of ways. First, the neural origins of the internal construct of an internal representation of vertical alignment arise within cortical zones located in the temporal and parietal cortex (Lopez et al., 2011), an area shared with the neural foundations for analysis of dimensional magnitudes and the “approximate number system”; thought to provide the foundation of numeracy skills (Walsh, 2003). Secondly, Witkin et al. (1977) suggest that field a independent mode in the perception of upright is associated with competence in restructuring the spatial field and associated with an articulated body concept. Factor analytical studies have also found PV loads significantly with activities associated with spatial restructuring (Witkin and Goodenough, 1981). Further, evidence supports the association between spatial restructuring and numeracy skills (Evans et al., 2013).

The moderate to strong relationships found in correlation and hierarchical regression, as well as between classification as having a field independent perceptual style and numeracy, suggest a link with STEM strands of academic tasks. The current study confirms previous findings, suggesting that children who accurately estimate vertical alignment are likely to perform at a higher level and may subsequently specialize in STEM fields (Witkin et al., 1975, 1977; Kent-Davis and Cochran, 1989; Evans et al., 2013).

However, being field independent is also an important advantage in reading, writing and for combined academic ability (overall academic scores), thus supporting the view that the current CRAFT methodology taps into cognitive processes involved in elements of general intelligence, as indicated by previous literature (Goodenough and Karp, 1961; Blowers and O’Connor, 1978; Berger and Goldberger, 1979; Agathos et al., 2015). In addition, the current findings add weight to the Dubois and Cohen (1970) proposal that the rod and frame test provides an analysis of a characteristic of intelligence “not contaminated by complex spatial reasoning,” and also support the view that general academic performance is predicted by the field independent classification, with no academic advantage in being field dependent.

Because eleven year old children develop from being relatively field dependent to relatively more field independent, it is possible that this transition period is particularly susceptible to a small visual angle rod and frame test. Dubois and Cohen (1970) conducted a study on 143 female undergraduate university students and found field independent participants out-performed field dependent participants and had a proclivity for STEM subjects. Unfortunately, the visual angle used in the above study was not provided, making it difficult to draw further conclusions.

Correlation analysis provided evidence of a significant but small association between CRF and PA with a number of academic variables. However, in the present study, elevated levels of CRF and PA were significantly associated with higher overall academic scores and writing score. Enhanced numeracy scores associated with higher levels of PA (*d* = 0.48) and CRF (*d* = 0.29) were the most significant; with no significant association found for reading, writing or overall academic scores. Support for the finding of an association between CRF solely with numeracy comes from previous research (Chaddock-Heyman et al., 2015; Huang et al., 2015). It appears that substantially increased levels of CRF are required before academic results in numeracy are positively influenced, a finding supported by previous research (Sibley and Etnier, 2003; Etnier et al., 2006; Drollette et al., 2014; Chaddock-Heyman et al., 2015)

The association between spatial processing ability of PV with numerical cognition may provide support for theories of embodied cognition (Koziol et al., 2012) which may well have evolved as an exaptive trait in the evolution of arboreal pre-humans, in which upright orientation was formulated from highly unpredictable compliant body support (on tree branches) in a visually complex and ambiguous environment made up of geometric mechanical shapes and forms; unstable and unreliable as a sensory frame of reference – unlike our modern terrestrial based world (Haynes et al., 2017). This theory builds on the proposals firstly by Thorpe et al. (2007) suggesting the arboreal origins of human uprightness; and by Godfrey-Smith (2002) arguing there is an association between environmental complexity and cognition; and proposals by Povinelli and Cant (1995) who contend arboreal requirements of large-bodied primates require complex sensorimotor adaptations that have led to self-awareness and other cognitive traits. Further, the “Dual Process Theory” (Ebenholtz and Glaser, 1982; Coren and Hoy, 1986) Ebenholtz (1977) may help explain the evolutionary construction differentiating small and large visual angle spatial perceptions. Engaging ventral (foveal) visual streams with small visual angle SVV possibly evolved in pre-human primates for upright search, fixation and identification of food sources embedded within the complex geometric arboreal field, with hand alignments to hold branches to support upright orientation. Large visual angle SVV tasks may have evolved to exploit peripheral (dorsal stream) visual systems designed for spatial orientation framed along the gravitational axis when the pre-human primate navigated and negotiated complex three dimensional spatial geometric environments on non-direct pathways containing gaps and obstacles. Pre-human primates may have then used many non-upright postures with spatial memory abilities to return to the nesting colony.

### Limitations

Necessarily, the cross-sectional design of the present study does not allow causal relations to be determined between SVV and STEM performance. Further, the pedometer measurements employed here are broad measures of PA and do not measure activity style or intensity. Some PA data collection technologies are more sensitive to the intensity load and style of PA, but these were not available to this study, and this constitutes a limitation. Finally, it is possible the instructions given to children in the conduct of the rod and frame test may have affected the study outcomes. However, the use of the rocket ship analogy and the time spent in describing and practicing the test before measurements were taken were consistent features throughout.

## Conclusion

The current study provides evidence that error scores from a small visual angle CRAFT are more strongly related to academic test results than are PA and CRF variables. Associations were found between the accuracy in predicting gravitational vertical and academic performance, particularly numeracy. Current results support the view that the existing methodology provides an important indicator of STEM potential. In terms of style of spatial processing, field independent children, compared to field dependent children, had better academic results with numeracy, exhibiting a strong effect size and emphasizing FI as a gauge of STEM potential. Field independent participants also had increased PA and CRF levels (small to moderate effect size). Finally, enhanced levels of CRF and PA (based on quartile splits) compared to lower levels of CRF and PA showed a significant small to moderate effect size in performance on the overall academic scores, numeracy and writing tests.

Whilst this is a cross-sectional analysis and no causal relationships can be attributed, the strength of the results provides grounds to suggest that possible PA pathways in intervention activities focusing on small scale visuo-perceptual tasks could influence academic performance. Supporting this view, recent evidence suggests that both motor activities (Adams et al., 2014) and musical training (Norton et al., 2005) can improve spatial cognition. A recent meta-review concluded that motor performance in combat sports, musical instrument studies and gymnastics (but not ballet) had the greatest impact on spatial abilities (Voyer and Jansen, 2017). Further, Newcombe (2017) proposes that spatial ability is malleable and suitable training may enhance STEM abilities. Future studies could therefore examine physical activities in pre-pubescent children who were engaging in spatial perception in both small and large field environments (small and large visual angles) to stimulate perceptual switching abilities, navigation and cognitive processing.

The strength of the findings here raises questions about the types of PA interventions that may influence vertical perception ability, and consequently academic results, and are scope for future research. For example, play environments with off-the-ground beam-like stepping bridges with rope hand supports forming complex geometric shapes to hold and orientate toward (stimulating small and large visual angles) are worthy of further investigation. Older children may benefit from single path map reading orienteering in a complex natural environmental setting combining both large and small field environments spatial perceptions with moderate to high levels of PA intensity. Further, elements of physical activities designed to stimulate alignment and orientation to vertical should logically include short periods of PA requiring moderate to high levels of intensity – for example soccer or tennis training drills engaging in small field visuo-spatial tasks, such as ball-at-feet soccer drills (Pesce, 2012) and yoga poses with visual focus and attention directed onto body parts (Diamond and Ling, 2016).

## Ethics Statement

This study was carried out in accordance with the recommendations of Australian Capital Territory Health and Community Care Human Research Ethics Committee. The protocol was approved by the Australian Capital Territory Health and Community Care Human Research Ethics Committee. All subjects gave written informed consent in accordance with the Declaration of Helsinki.

## Human Participants Approval Statement

This study was approved by the Australian Capital Territory Health and Community Care Human Research Ethics Committee.

## Author Contributions

WH was the lead author, designed the research, carried out the data analysis, and worked on the conceptual design. GW was involved in the authorship, data analysis, and statistical modeling. RA contributed to the statistical design, data analysis, and paper construction. BI contributed to the research design and paper construction.

## Conflict of Interest Statement

The authors declare that the research was conducted in the absence of any commercial or financial relationships that could be construed as a potential conflict of interest.

## Footnotes

1 https://www.nap.edu.au/
